# Highly Variable *Streptococcus oralis* Strains Are Common among Viridans Streptococci Isolated from Primates

**DOI:** 10.1128/mSphere.00041-15

**Published:** 2016-03-09

**Authors:** Dalia Denapaite, Martin Rieger, Sophie Köndgen, Reinhold Brückner, Irma Ochigava, Peter Kappeler, Kerstin Mätz-Rensing, Fabian Leendertz, Regine Hakenbeck

**Affiliations:** a Department of Microbiology, University of Kaiserslautern, Kaiserslautern, Germany; b Project Group 3 Epidemiology of Highly Pathogenic Microorganisms, Robert Koch-Institute, Berlin, Germany; c Behavioral Ecology and Sociobiology Unit, German Primate Center, Göttingen, Germany; Swiss Federal Institute of Technology, Lausanne, Switzerland

**Keywords:** *Streptococcus oralis*, horizontal gene transfer, primates, teichoic acid, viridans streptococci, virulence factors

## Abstract

Viridans streptococci were obtained from primates (great apes, rhesus monkeys, and ring-tailed lemurs) held in captivity, as well as from free-living animals (chimpanzees and lemurs) for whom contact with humans is highly restricted. Isolates represented a variety of viridans streptococci, including unknown species. *Streptococcus oralis* was frequently isolated from samples from great apes. Genotypic methods revealed that most of the strains clustered on separate lineages outside the main cluster of human *S. oralis* strains. This suggests that *S. oralis* is part of the commensal flora in higher primates and evolved prior to humans. Many genes described as virulence factors in *Streptococcus pneumoniae* were present also in other viridans streptococcal genomes. Unlike in *S. pneumoniae*, clustered regularly interspaced short palindromic repeat (CRISPR)–CRISPR-associated protein (Cas) gene clusters were common among viridans streptococci, and many *S. oralis* strains were type PI-2 (pilus islet 2) variants. *S. oralis* displayed a remarkable diversity of genes involved in the biosynthesis of peptidoglycan (penicillin-binding proteins and MurMN) and choline-containing teichoic acid. The small noncoding *cia*-dependent small RNAs (csRNAs) controlled by the response regulator CiaR might contribute to the genomic diversity, since we observed novel genomic islands between duplicated csRNAs, variably present in some isolates. All *S. oralis* genomes contained a β-*N*-acetyl-hexosaminidase gene absent in *S. pneumoniae*, which in contrast frequently harbors the neuraminidases NanB/C, which are absent in *S. oralis*. The identification of *S. oralis*-specific genes will help us to understand their adaptation to diverse habitats.

**IMPORTANCE** *Streptococcus pneumoniae* is a rare example of a human-pathogenic bacterium among viridans streptococci, which consist of commensal symbionts, such as the close relatives *Streptococcus mitis* and *S. oralis*. We have shown that *S. oralis* can frequently be isolated from primates and a variety of other viridans streptococci as well. Genes and genomic islands which are known pneumococcal virulence factors are present in *S. oralis* and *S. mitis*, documenting the widespread occurrence of these compounds, which encode surface and secreted proteins. The frequent occurrence of CRISP-Cas gene clusters and a surprising variation of a set of small noncoding RNAs are factors to be considered in future research to further our understanding of mechanisms involved in the genomic diversity driven by horizontal gene transfer among viridans streptococci.

## Abstract (precis)

*Streptococcus pneumoniae* is a rare example of a human-pathogenic bacterium among viridans streptococci, which consist of commensal symbionts, such as the close relatives *Streptococcus mitis* and *S. oralis*. We have shown that *S. oralis* can frequently be isolated from primates and a variety of other viridans streptococci as well. Genes and genomic islands which are known pneumococcal virulence factors are present in *S. oralis* and *S. mitis*, documenting the widespread occurrence of these compounds, which encode surface and secreted proteins. The frequent occurrence of CRISP-Cas gene clusters and a surprising variation of a set of small noncoding RNAs are factors to be considered in future research to further our understanding of mechanisms involved in the genomic diversity driven by horizontal gene transfer among viridans streptococci.

## INTRODUCTION

Viridans streptococci are a major part of the commensal microbiota of the upper respiratory tract of humans (for a review, see reference 1). Only one member, *Streptococcus pneumoniae*, represents a major pathogen causing a variety of diseases, such as pneumonia, otitis media, sepsis, and meningitis, whereas even its closest relatives *Streptococcus mitis*, *Streptococcus oralis*, and the recently described *Streptococcus pseudopneumoniae* (2) are rarely associated with disease. Genomic analyses represent a powerful tool to further our understanding of the genetic basis for the pathogenic potential of *S. pneumoniae* and to decipher the evolutionary relationship between these species.

Many gene products have been described as virulence factors in *S. pneumoniae*. Strains carrying mutations in these genes are less virulent in mouse models. However, most of them are probably required for host interaction rather than being directly responsible for disease since in most cases homologs are present in *S. pneumoniae*’s nonpathogenic commensal relatives *S. mitis* and *S. pseudopneumoniae* (2–4). There are only a few components that are clearly associated with *S. pneumoniae* and which are not or are only rarely found in commensal streptococci: the pneumolysin Ply, a pore-forming toxin whose gene is located on an islet together with the autolysin gene *lytA*, and the three choline-binding proteins (CBPs) PspA, PcpA, and PspC, including the Hic variant of Psp and the hyaluronidase HlyA. Moreover, the biochemically highly diverse capsule is essential for pneumococcal pathogenicity. Nevertheless, even those compounds are either absent (rare) in *S. pneumoniae* or highly varied. A random distribution of virulence genes (*lytA*, *ply*, and the *cap* locus, representing the capsule biosynthesis operon) has been observed among *S. mitis* strains (5), suggesting that the acquisition and loss of genes is an ongoing process in this group of bacteria. It has been proposed that the three species *S. mitis*, *S. pneumoniae*, and *S. pseudopneumoniae* arose from an ancient bacterial population that included all *S. pneumoniae*-specific genes (6), and genomic analysis of 35 *Streptococcus* spp. indicated that the common ancestor was a pneumococcus-like species (5). Moreover, the authors provided evidence that interspecies gene transfer occurred mainly unidirectionally from *S. mitis* to *S. pneumoniae*.

Due to the ability of streptococci to develop genetic competence resulting in a large accessory genome, identification to the species level has been problematic using phenotypic and physiological criteria. Therefore, a variety of genotypic methods have been established based on sequences from the core genes (housekeeping genes). Multilocus sequence typing (MLST) has become the gold standard for clonal analysis, especially within species (7), and multilocus sequence analysis (MLSA) has been applied to differentiate closely related streptococcal species (8). Nonetheless, recombinogenic bacteria do not form clusters with clear boundaries and have been termed “fuzzy species” (9). Phylogenetic analyses show that *S. pneumoniae* is a single lineage in a cluster formed by a variety of *S. mitis* clusters, whereas the *S. oralis* cluster is well separated (5, 6, 10). Diversification within the *S. pneumoniae* lineage has probably occurred during growth of the human population, its primary host (5). This suggests that the diversification of *S. mitis* and *S. oralis* took place earlier and that great apes might still harbor *S. mitis* and *S. oralis* as part of their commensal flora.

We therefore investigated the distribution of viridans streptococci among great apes and other primates. Samples were obtained from animals held in captivity, namely, gorillas, orangs, and bonobos from the Frankfurt Zoo and rhesus monkeys and ring-tailed lemurs (*Lemur catta*) from the German Primate Center, as well as from free-living animals for whom contact with humans is highly restricted, namely, chimpanzees from the Taï National Park, Ivory Coast, lemurs from the Kirindy Forest in Madagascar (Verreaux’s sifaka, *Propithecus verreauxi*), red-fronted lemurs (*Eulemur rufifrons*), Western fat-tailed dwarf lemurs (*Cheirogaleus medius*), and gray mouse lemurs (*Microcebus murinus*). In the first part of our study, the species are described based on genotypic methods. *S. oralis* was common among great apes, including wild chimpanzees, and was also found in rhesus monkeys. In the second part of our study, the genomes from 23 isolates are analyzed in detail. Emphasis is placed on the presence of large genomic islands which are part of the accessory genome of *S. pneumoniae*, small noncoding RNAs controlled by a highly conserved two-component system, CiaRH, *S. pneumoniae* virulence factors, and genes involved in peptidoglycan and teichoic acid (TA) metabolism.

## RESULTS

### Determination to the species level of viridans isolates from primates by MLSA.

All isolates from primates were initially characterized for their morphologies (by colony and microscopic analysis of cells), antibiotic susceptibilities, 16S rRNAs, and in some cases SDS-PAGE protein patterns of cell lysates and penicillin binding protein (PBP) profiles. A total of 139 isolates was included in further analyses (see Table S1 in the supplemental material). MICs of oxacillin above 1 µg/ml and resistance to antibiotics of other classes were common among isolates from animals held in captivity, but isolates from free-living chimpanzees of the Taï National Park in Africa did not show significant resistance patterns for any of the antibiotics tested (Table S1). If a group of isolates obtained from one animal showed properties identical to those listed above, only one isolate was subjected to further analysis by MLST. This group consisted of 44 isolates, including all isolates from Taï chimpanzees and at least three isolates from the other group of animals. Most isolates from zoo animals were furthermore defined by MLST analysis (see below).

10.1128/mSphere.00041-15.4 Table S1  Isolates of primates, genotypic characterization, and antibiotic resistance profile. Download Table S1, XLSX file, 0.04 MB. Copyright © 2016 Denapaite et al. 2016 Denapaite et al. This content is distributed under the terms of the Creative Commons Attribution 4.0 International license.

**FIG 1:**
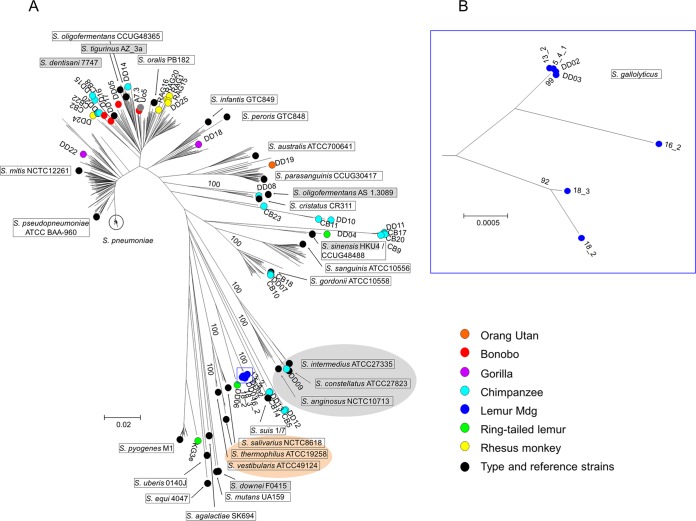
MLSA trees of strains from this study and reference strains. (A) A neighbor-joining tree was constructed using the concatenated sequences of the MLSA loci from 44 strains of this study combined with sequences of 427 strains from the study of Bishop et al. (8). In addition, MLSA genes were extracted from the genomes of *S. tigurinus* AZ_3a (GCF_000344275.1), *S. dentisani* 7747 (GCF_000382805.1), *S. sinensis* HKU4 (GCF_000767835.1), *S. oligofermentans* AS1.3089 (CP004409.1), and *S. downei* F0415 (GCA_000180055.1) (strains included in addition to those from the study by Bishop et al. [8] are shaded gray). Viridans group reference and type strains are framed. The Anginosus group of viridans streptococci is shaded gray and the Salivarius group orange. The color key for reference strains and isolates from primates is indicated on the right. Mdg, Madagascar. (B) Neighbor-joining tree of the *Streptococcus* sp. cluster of strains from Madagascar lemurs (blue circles in the square in panel A) magnified to show more clearly the clustering of the strains. Bootstrap values (percentages) are based on 1,000 replications. The bar refers to genetic divergence as calculated by the MEGA software.

Figure 1 shows a neighbor-joining radial tree of the primate isolates in comparison with the published MLSA data set of a wide variety of streptococcal species (8). In addition, MLSA data were extracted from another five genomes available recently. *Streptococcus oligofermentans* AS1.3089 (11) was positioned within the Anginosus group, as expected, whereas the reference strain used by Bishop et al. (8), *S. oligofermentans* CCUG48365, clustered within *S. oralis*, strongly suggesting that the latter strain has been misidentified as suggested before (8). *Streptococcus sinensis* HKU4 (12) was located at nearly the same position as *S. sinensis* SK1972 (8), and *Streptococcus downei* F0415 was part of the Mutans group. Moreover, *Streptococcus tigurinus* AZ_3a and *Streptococcus dentisani* 7747, representing two new species that have been described as being closely related to *S. oralis* (13–15), were located within the *S. oralis* group (Fig. 1) and will be discussed below. The primate isolates covered a wide range of *Streptococcus* spp. Sixteen isolates obtained from zoo animals, wild chimpanzees, and rhesus monkeys were found among the *S. oralis* cluster. One isolate from a gorilla (DD22) clustered among *S. mitis* strains, and one from another gorilla (DD18) clustered close to *Streptococcus infantis*/*Streptococcus peroris*. Three isolates from Taï chimpanzees were defined as *Streptococcus gordonii* (CB10, CB18, and DD07), DD08 was defined as *Streptococcus cristatus*, and DD04 from ring-tailed lemurs mapped close to *S. sinensis*, all of which are within the Mitis group of viridans streptococci. One isolate (DD09) from a chimpanzee was defined as *Streptococcus constellatus*, which belongs to the Anginosus group of viridans streptococci. No streptococci could be isolated from free-living lemurs of Madagascar, where *Enterococcus* sp. and *Lactococcus* sp. were commonly obtained. Only from *Propithecus verreauxii* were we able to obtain *Streptococcus* sp. that could not be defined by MLSA (Table S1).

A total of 20 isolates fell into different lineages outside the streptococcal species included in the MLSA tree (Fig. 1). A cluster of seven isolates from Madagascar lemurs is magnified in Fig. 1B. Some isolates could not be identified to the species level by MLSA. From Taï chimpanzees, there were two clusters within the Mitis group consisting of three strains (CB23, CB11, and DD10) and four strains (DD11, CB9, CB17, and CB20) and two pairs of strains (DD12/CB5 and DD13/CB14) between the Anginosus and Salivarius groups that could not be identified to the species levels; from ring-tailed lemurs, there were two isolates between the Salivarius group and *S. pyogenes* (KG3e and DD06) that could not be identified to the species level. Comparison of their 16S rRNA sequences did not match any known sequences from the NCBI data bank except those of DD06 (*Streptococcus lutetiensis*) and the Madagascar lemurs (*Streptococcus gallolyticus*) (Fig. 1B). It should be noted that 16S rRNA sequences pose significant problems for identification, and matches below 100% are not very meaningful to differentiate between species (see reference 16 and references therein).

### Determination to the species level by genome analysis and plasmids.

Twenty-five streptococcal isolates (named “DD” followed by consecutive numbers) were chosen for whole-genome sequencing, 23 of which were *Streptococcus* spp. (see Table S1 in the supplemental material). This included 8 *S. oralis* isolates representing different lineages of the MLSA tree to cover a broad range of variation within this species. In addition, we used 1 isolate each of *S. gordonii*, *S. cristatus*, *S. constellatus*, *S. infantis*, and *S. mitis* as defined by MLSA and 10 isolates of unclear species determination according to MLSA.

The species defined by MLSA were confirmed by genome sequences (Table S2). According to BLAST analysis with 16S rRNA, MLSA genes, and *pbp2a* in the NCBI microbial genome data bank (Table S3), the two genomes of DD02 and DD03 from Lemur isolates were identified as *S. gallolyticus*, DD06 from ring-tailed lemurs was identified as *S. lutetiensis*, and DD19 from zoo animals was identified as *S. parasanguinis*. DD04 was close to *S. sinensis*. There remained four isolates from Taï chimpanzees (DD10, DD11, DD12, and DD13) whose species could not be determined; for none of their genes did we find close matches in the NCBI data bank.

10.1128/mSphere.00041-15.5 Table S2  Presence of selected genes and gene clusters in the genomes. Download Table S2, XLSX file, 0.1 MB. Copyright © 2016 Denapaite et al. 2016 Denapaite et al. This content is distributed under the terms of the Creative Commons Attribution 4.0 International license.

10.1128/mSphere.00041-15.6 Table S3  Determination of the species of isolates not determined by MLSA. Download Table S3, XLSX file, 0.01 MB. Copyright © 2016 Denapaite et al. 2016 Denapaite et al. This content is distributed under the terms of the Creative Commons Attribution 4.0 International license.

During preparation of chromosomal DNA, plasmids were detected in five samples. The plasmid of *S. oralis* strain DD25 from rhesus monkeys was identical to the *S. pneumoniae* pSpnP1 plasmid, large parts of which were also found in *S. oralis* strain DD24. Fragments related to pSpnP1 were also present in plasmids from *S. oralis* strain DD17, and *S*. *infantis* strain DD18 was partially related to a plasmid from *S. pseudopneumoniae* IS7493 pDRPIS7493. No significant matches to the *S. gallolyticus* strain DD03 plasmid were found by BLAST analysis.

### A closer look at *S. oralis.*

**FIG 2:**
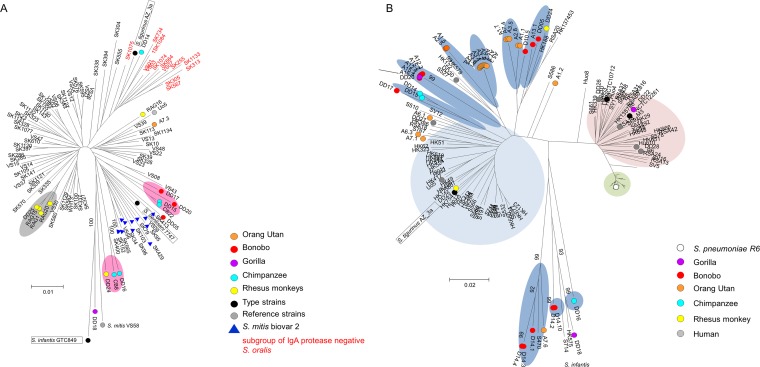
Phylogenetic trees of *S. oralis* and *S. mitis* strains. (A) Tree generated by MLSA loci of *S. oralis* from this study and sequences of *S. oralis* strains, *S. tigurinus* AZ_3a, and *S. dentisani* 7747 from the MLSA tree shown in Fig. 1. Gray shading, cluster of isolates from rhesus monkeys; pink shading, lineages consisting of primate isolates only. One *S. mitis* strain from the study of Bishop et al. (8), VS58, was included for comparison. Red letters indicate the subgroup of IgA protease-negative *S. oralis* isolates specified in reference 8. Bootstrap values (percentages) are based on 1,000 replications. (B) Phylogenetic relationship of primate isolates, including 38 isolates from zoo animals determined by MLST (7) but excluding *ddl*, combined with sequences of 119 human isolates (*S. oralis*, *S. mitis*, and *S. pneumoniae*) from different geographic locations (10) (isolate numbers are preceded by Hu for Hungary, RSA for South Africa, S for Spain, HK for Hong Kong, and B for Germany). Lineages within the *S. oralis* cluster containing only isolates from primates are shaded in dark blue. Bootstrap values (percentages) are based on 500 replications. The bar refers to genetic divergence as calculated by the MEGA software.

The published MLSA data set distinguishes three phenotypically distinct subclusters among *S. oralis* strains: one which covered strains previously defined as *S. mitis* biovar 2, an IgA protease-negative *S. oralis* cluster, and various lineages of IgA protease-positive *S. oralis* (8). As can be seen in Fig. 2A, S was found within the *S. oralis* subcluster of IgA protease-negative strains and *S. dentisani* among the subcluster of strains previously defined as *S. mitis* biovar 2 (8). We found only one isolate from primates within the biovar 2 group (DD05 from a bonobo) and one within the IgA protease-negative group (DD14) (Fig. 2A). Four isolates from rhesus monkeys formed one subcluster (grey in Fig. 2A). Seven isolates were found on different lineages outside the main *S. oralis* group of human *S. oralis* strains (pink in Fig. 2A).

We then analyzed 38 isolates from zoo primates by MLST since most of them were suspected of being *S. oralis* based on the preliminary characterization. The MLST sequences extracted from the genomes of isolates from rhesus monkeys and chimpanzees were included. The results were compared to previously published MLST data (10) derived from a set of 119 *S. pneumoniae*, *S. mitis*, and *S. oralis* isolates from different geographic areas, and the MLST sequences from *S. tigurinus* AZ_4a were also included (Fig. 2B) (not all MLST sequences from the genome of *S. dentisani* 7747 were included since the genes *spi* and *gdh* were not found in the genome). The presence of multiple subclusters within the *S. oralis* cluster is evident also in the MLST-based phylogeny shown here, which positions *S. tigurinus* within the main *S. oralis* cluster. Again, most *S. oralis* isolates from primates except four (A7.1, A6.3, and A6.1 from zoo apes and DD25 from a rhesus monkey) were located outside the main cluster of human *S. oralis* strains in several lineages (blue in Fig. 2B). In only one case were identical MLST sequences obtained from strains from two different primate species: DD20 from a gorilla, A12.3 from bonobo Ku, and A15.2/A15.3 from bonobo Zo (arrow in Fig. 2B; see Table S1 in the supplemental material). DD18, which was defined by MLSA as *S. infantis*, and DD22, identified as *S. mitis*, were positioned in the tree outside the *S. oralis* lineages.

Thus, MLSA as well as MLST data showed that most *S. oralis* isolates from primates—independently of whether they had been obtained from animals held in captivity or from free-living animals—were distinct from those of the many human isolates from different geographic areas, including China, South Africa, and several eastern and western European countries.

### Dissemination of large genomic islands.

**FIG 3:**
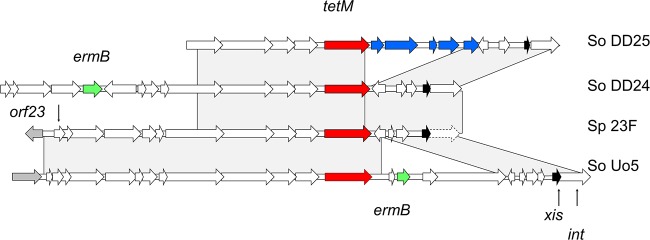
Comparison of TetM-containing genetic elements in two genomes from rhesus monkeys with those of *S. pneumoniae* Spain 23F-1 (Sp 23F) and *S. oralis* Uo5 (So Uo5). Red, *tetM*; green, *ermB*; blue, homology to *Enterococcus faecium* plasmid pM7M2 genes. *xis* is the excisionase gene (black); the integrase is a pseudogene in *S. pneumoniae* (dashed arrow). Gray areas indicate BLASTn matches between sequences.

In the following analyses, we included another three human *S. oralis* and four *S. mitis* isolates described before (10, 17). Two isolates from rhesus monkeys (*S. oralis* strains DD24 and DD25) were tetracycline resistant; DD24 was erythromycin resistant as well. Tetracycline resistance in *S. pneumoniae* is most commonly conferred by *tetM*, located on an integrative conjugative element (ICE) of the Tn*916* family (18, 19). As shown in Fig. 3, both genomes carried parts of Tn*916* like the *S. pneumoniae* Spain 23F-1 clone (20). *S. oralis* strain DD24 contained *ermB* located on an ICE which is also present in a variety of *Streptococcus* sp. genomes, including that of *S. pneumoniae* Hungary 19A-6. In contrast, the human isolate *S. oralis* Uo5 contained *ermB* next to *tetM*, a genotype frequently also found in *S. pneumoniae* (18). The *S. oralis* strain DD25 Tn*916* region had an insert corresponding to *Enterococcus faecium* plasmid pM7M2 sequences (21) which are present in a wide variety of Gram-positive bacteria, including *Staphylococcus* spp., *Bacillus* spp., and *Streptococcus* spp., according to BLAST analysis with the NCBI nucleotide data bank (blue in Fig. 3).

There are several large gene clusters of the accessory genome in *S. pneumoniae* (>10 kb) implicated in modulation of the pathogenicity potential (22) and which are found to be widespread among different species. One cluster harbors genes encoding a serine-rich cell surface protein (named PsrP in *S. pneumoniae* and MonX in *S. mitis* B6) with accessory components responsible for glycosylation and export. Serine-rich proteins are adhesins common among Gram-positive bacteria and contribute to a variety of diseases (for a review, see reference 23). This cluster was widespread also among the primate genomes (Table S2). Moreover, a region containing genes for a V-type ATPase was present in several primate genomes (Table S2).

**FIG 4:**
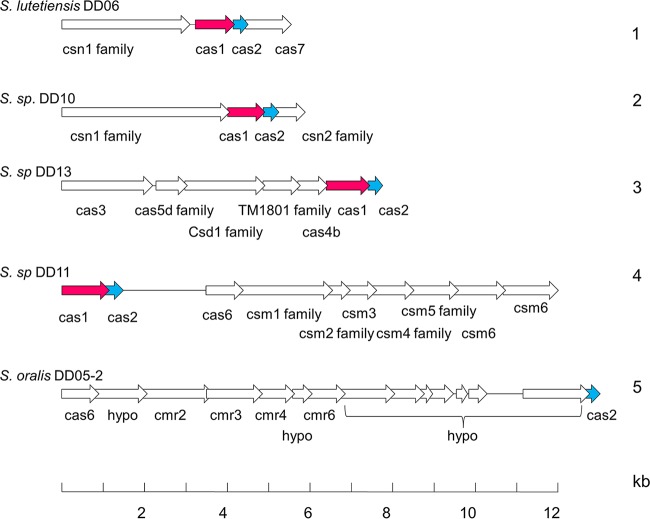
CRISPR-Cas gene clusters in streptococcal genomes. The five classes (designated 1 to 5) of CRISPR-Cas gene clusters identified in the genomes of this study are shown; representative genomes containing these clusters are indicated on the left. The annotation given by the RAST server (http://rast.nmpdr.org/) was used. Red, Cas1 genes; blue, Cas2 genes; *S. sp*, Streptococcus sp.

CRISPR-Cas (*c*lustered *r*egularly *i*nterspaced *s*hort *p*alindromic *r*epeats–*C*RISPR-*as*sociated proteins) loci represent defense systems against foreign genetic elements. Although *S. pneumoniae* does not contain CRISPR sequences, they were found among *S. mitis* and *S. oralis* isolates (5), but information concerning other streptococcal species is still limited (24). We detected CRISPR-Cas gene clusters in most of the streptococcal genomes, and several genomes contained more than one CRISPR-Cas cluster at different genomic positions (Table S2). Five different cluster arrangements were observed (examples are shown in Fig. 4); *S. oralis* genomes contained clusters of types 1, 2, and 5. Four of these clusters contain Cas1 genes which clustered according to their genomic arrangement (Fig. S1), as described by Makarova et al. (24).

10.1128/mSphere.00041-15.1 Figure S1  Phylogenetic tree of Cas1 proteins in streptococcal genomes used in this study. The number refers to the different CRISPR-Cas clusters shown in Fig. 3. p indicates partial sequences. The tree was generated with MEGA6 using Clustal alignment. The bar refers to genetic divergence as calculated by the MEGA software. Download Figure S1, TIF file, 0.2 MB. Copyright © 2016 Denapaite et al. 2016 Denapaite et al. This content is distributed under the terms of the Creative Commons Attribution 4.0 International license.

**FIG 5:**
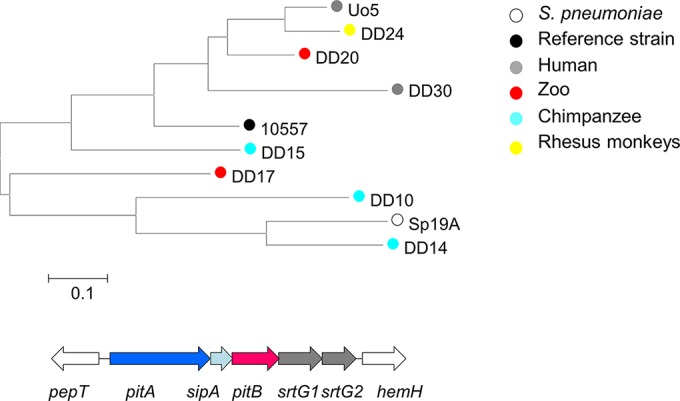
Comparison of PitB genes deduced from pilus clusters in streptococcal genomes. The phylogenetic tree was generated with MEGA6 using the Clustal alignment. PitB of *S. pneumoniae* SpTCH8431/19A and *S. oralis* ATCC 10557 represent the closest tBLASTn matches to PitB from *S. oralis* Uo5. The pilus cluster of *S. oralis* Uo5 is shown below. Red, *pitB*. The bar indicates changes per nucleotide position. *pitA* and *pitB*, pilus proteins; *sipA*, essential for pilin biosynthesis; *srtG1* and *srtG2*, sortases. White genes mark conserved flanking genes outside the pilus cluster.

An interesting case of intra- and interspecies gene transfer events among the Mitis group of streptococci is the presence of new variants of pilus islet 2 (PI-2), described to occur in *S. oralis*, *S. mitis*, and *S. sanguinis* (25). PI-2 pili are present in a limited number of *S. pneumoniae* strains (26, 27) and facilitate adhesion to eucaryotic cells. A PI-2 islet is present in *S. oralis* Uo5, and we used the deduced PitB protein, the major pilus subunit, to screen the primate genomes for the presence of pilus variants. Six primate genomes contained *pitB*-related genes, five *S. oralis* genomes were from a variety of primates, and one was of unclear species. All of them encoded PitB variants distinct from that of the reference strain *S. oralis* ATCC 10577 used by Zähner et al. (25) (Fig. 5). All these data indicate that interspecies gene transfer is a common feature among viridans streptococci independently of the source of isolation; alternatively, the genes have been lost in some of the strains.

In further analyses, we concentrated on cell surface components, namely, genes encoding enzymes for peptidoglycan and teichoic acid biosynthesis, cell surface proteins, and virulence factors not detected in *S. mitis* B6 (3) or *S. oralis* Uo5 (28), since they are the major factors responsible for the interaction with host cells.

### Penicillin-binding proteins and MurMN.

*S. pneumoniae* contains six PBPs (the bimodular transglycosylase/transpeptidases PBP1a, -1b, and -2a, the transpeptidases PBP2x and -2b, and the d,d-carboxypeptidase [CPase] PBP3), and homologs to all six PBPs were present in the streptococcal genomes of this study. Resistance to β-lactam antibiotics is due to alterations in at least three PBP genes, PBP2x, PBP2b, and PBP1a, which are known to be encoded by mosaic genes in resistant *S. pneumoniae*, *S. mitis*, and *S. oralis* strains (for a review, see reference 29). No mosaic structures have been detected so far in PBP2a, which is only occasionally involved in strains of high-level resistance, and PBP3 is not known to contribute to resistance in clinical isolates of *S. pneumoniae*.

**FIG 6:**
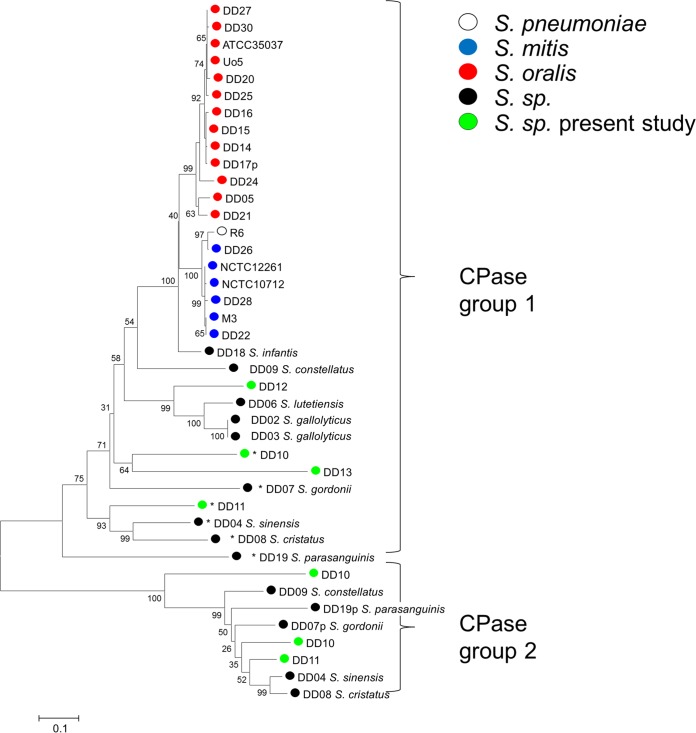
Distribution of PBP3 homologs in streptococcal genomes. A phylogenetic tree was constructed from deduced protein sequences from PBP3 d,d-carboxypeptidases using the MEGA6 software and muscle alignment. Proteins with at least one unusual active-site motif, indicating a nonfunctional PBP, are marked by asterisks, and partial sequences indicated by *P* bootstrap values (percentages) are based on 1,000 replications. The bar refers to genetic divergence as calculated by the MEGA software.

Genes encoding all six PBPs were found in all genomes analyzed here. Surprisingly, more than one CPase homolog was found in several of the streptococcal genomes (*S. constellatus*, *S. parasanguinis*, *S. gordonii*, *S. sinensis*, and *S. cristatus*), which formed two well-separated homology clusters (group 1 and group 2 in Fig. 6). The larger group, group 1, which roughly reflects the phylogeny of the species, consisted of the common PBP3 homolog present in all genomes, as expected for a gene product of the core genome, whereas this is less obvious for group 2 CPases. All group 2 CPases contained the active-site motifs SMSK, SSN, and KTG; *Streptococcus* sp. strain DD10 even contained a second group 2 protein with the deduced motifs SMAK, SSA, and KTG. This indicates that all group 2 proteins are functional enzymes. In contrast, in almost all strains with group 2 CPases, with the exception of *S. constellatus* strain DD09, the group 1 CPases had mutations at the active-site serine and/or at the conserved lysine residue within the SXXK motif (strains with this motif are marked with an asterisk in Fig. 6), suggesting an inactive enzyme. In all genomes where a group 2 CPase was present, the PBP3 homolog was positioned between the SufF gene and a gene encoding an ABC transporter. In contrast, group 2 CPases were found at three different genomic environments, indicating that they were acquired later during evolution.

The three PBPs known to be related to the resistance phenotype (PBP2x, PBP2b, and PBP1a) were examined more closely. The aims were to see how variable PBP sequences are among the *S. oralis* isolates, whether PBP sequences are shared between PBP genes from human and primate isolates, and whether signs of gene transfer are detectable.

**FIG 7:**
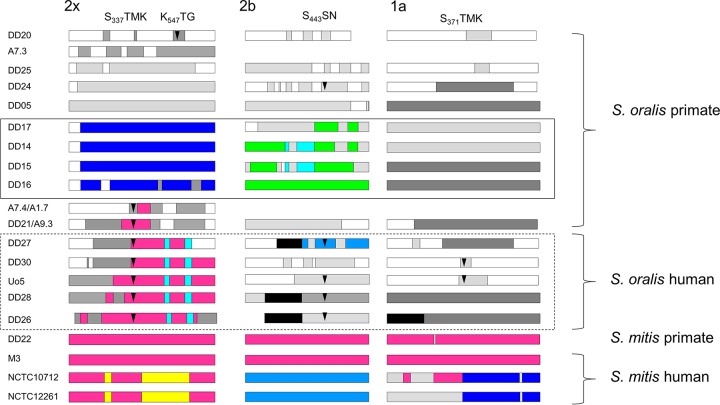
Mosaic PBPs in *S. mitis* and *S. oralis*. Mosaic gene structures were deduced by comparison to *S. oralis* ATCC 35037 PBP genes. Sequences that are highly similar to each other (<5% difference) are shown in the same color; sequences of different colors diverge from each other by at least 15%. Light gray, divergence from *S. oralis* ATCC 35037 of approximately 5%; dark gray, divergence by >15%; arrowheads, mutations within or close to active-site motifs which are shown on top; solid-line box, PBPs from free-living chimpanzees; dashed box, PBPs from human isolates with high-level penicillin resistance. The amino acid numbers are those of PBPs from sensitive *S. pneumoniae* strains.

Mosaic structures were apparent in penicillin-sensitive isolates from primates compared to the penicillin-sensitive strain *S. oralis* ATCC 35037 (Fig. 7), in agreement with the high variability of PBP genes detected in a large number of human commensal streptococci (30). Interestingly, the sequences from the three genes obtained from Taï chimpanzees were distinct from those of all other PBP genes; BLAST searches of sequences in the NCBI data bank also did not reveal any identical genes. This confirms that these isolates belong to a special group of *S. oralis* strains that has evolved independently. Moreover, the primate isolates clustered separately from the human isolates, with the exception of *S. oralis* strain DD20 (bonobo isolate), which was closely related to ATCC 35037, and *S. mitis* strain DD22 (gorilla isolate), which carries PBP genes almost identical to *S. mitis* M3 genes (Fig. 7 and Fig. S2). These data also clearly indicate that there is no correlation between PBP2x, PBP2b, and PBP1a sequences; PBP2x from *S. oralis* strains DD14, DD15, and DD17 were identical, and all PBP2b and PBP1a sequences differed from each other (Fig. S2).

10.1128/mSphere.00041-15.2 Figure S2  Phylogenetic trees of PBPs from *S. mitis* and *S. oralis*. Light-blue area, *S. oralis*; dark-blue area, *S. oralis* with high-level resistance; pink area, *S. mitis*. *S. pneumoniae* R6 was used for comparison. Trees were generated with MEGA6 using Clustal alignment. The bar refers to genetic divergence as calculated by the MEGA software. Download Figure S2, TIF file, 0.5 MB. Copyright © 2016 Denapaite et al. 2016 Denapaite et al. This content is distributed under the terms of the Creative Commons Attribution 4.0 International license.

Mosaic structures were most obvious in PBP2x, PBP2b, and PBP1a genes from the human isolates with high-level penicillin resistance (Fig. 7). The mosaic PBP2x genes belonged to the major PBP2x family common among oral streptococci, with a large sequence block highly related to some *S. mitis* strains (10). The mosaic structures indicate multiple gene transfer events among different species.

Mutations known to contribute to the resistance phenotype (for a review, see reference 29) were detected only within or close to the active-site motifs. Seven PBP2x variants contained T338A, whereas PBP2x of *S. oralis* DD20 contained the mutation Q552E, consistent with the lower susceptibility to cefotaxime of the strain. PBP2b mutations were also common (T446A), and *S. oralis* DD30 contained the same PBP1a mutation as *S. oralis* Uo5 (T372S) (see the arrowheads in Fig. 7). The PBP2b mutation confers only a small increase in β-lactam MICs (31), and therefore it is not surprising that *S. oralis* DD24 containing this mutation expresses only marginal resistance. In all cases, the PBP mutations were located within mosaic blocks; i.e., they have been acquired by gene transfer and are thus most likely not spontaneous mutations. In summary, mosaic structures are common also in *S. oralis* PBP2x genes, not only in the resistant isolates but also among penicillin-sensitive strains.

PBPs use muropeptides as the substrates for their transpeptidation reaction and the formation of cross-links in the peptidoglycan. In *S. pneumoniae*, MurM and MurN enzymes, which are responsible for the synthesis of branched muropeptides, have been described. MurM adds an l-Ala or l-Ser to the ε-amino group of the l-Lys residue of lipid II, and MurN adds another l-Ala residue. The branched peptides are used as an acceptor substrate for the transpeptidation reaction of PBPs, resulting in interpeptide bridges in mature peptidoglycan (32). MurM genes have a mosaic structure in some penicillin-resistant *S. pneumoniae* strains (33) and are thus also the subject of gene transfer events. We recently showed that *S. oralis* Uo5 contains an unusual MurM gene and no *murN*, consistent with the presence of branched muropeptides containing only 1 alanine residue attached to lysine (34) instead of the Ala-Ala or Ser-Ala dipeptide found in *S. pneumoniae* (35–38). We therefore searched the genomes for the presence of *murMN* to see whether the lack of *murN* is a common feature of *S. oralis*.

Although MurMN was present in most streptococcal genomes (Table S2), the situation among the *S. oralis*/*S. mitis* group was surprisingly varied. We found MurM homologs only in two *S. oralis* genomes (DD17 and DD21) and not in the other *S. oralis* genomes, regardless of the MurM variant used in BLAST searches (MurM from *S. oralis* Uo5, *S. mitis* B6 or *S. infantis* DD19). Similarly, BLAST searches of *S. oralis* draft genomes in the NCBI data bank revealed only one genome which contained a MurM homolog. Also, the *S. mitis* genome DD22 contained *murM* within a genomic environment similar to that of *S. oralis* Uo5 but not *murN*, whereas other *S. mitis* genomes contained *murMN* in a genetic environment similar to that of *S. mitis* B6.

### TAs and choline-binding proteins.

The genomes were screened for genes required for teichoic acid (TA) backbone biosynthesis and decoration. The sequenced strains can be divided into two groups. The first group of 13 strains contains one gene whose product is the key enzyme LtaS, the lipoteichoic acid (LTA) synthetase which catalyzes the polymerization of type I LTA, containing a polyglycerolphosphate chain, the most frequently encountered cell wall polymer (39). This group includes different *Streptococcus* species (Table S2), and their entire LtaS proteins are similar to LtaS of *S. mitis* B6 (Smi0753), with 58% to 75% of their amino acids being identical to those of LtaS of B6. Three *S. mitis* strains (DD22, DD26, and DD28) contained a deduced LtaS protein with >96% identity to LtaS of B6. In contrast, *ltaS* homologs were not found in *S. oralis* and *S. infantis* genomes, in agreement with published data (40).

The 12 strains where *ltaS* was absent contained the genes involved in the biosynthesis of the unusually complex, choline-containing type IV LTA, typical for *S. pneumoniae* and closely related species. In *S. pneumoniae*, LTA and wall teichoic acid exhibit identical structures within their repeating units (RU) (41). In the *S. mitis* B6 strain, the TA gene content and genetic organization are nearly identical to those of *S. pneumoniae* R6, except that *S. mitis* may contain galactose instead of glucose in its TA repeating unit (40). In contrast, *S. oralis* Uo5 produces a structurally different TA repeating unit and has structural complexity even greater than that of pneumococcal LTA (42). Two *S. mitis* isolates, DD22 and DD28, contain *S. pneumoniae*-type TA biosynthesis clusters but differ in their glycosyl transferase genes, suggesting that DD22 contains glucose but that DD28 contains galactose in its TA. In contrast, all *S. oralis* isolates and *S. mitis* DD26 contain glycosyl transferase genes of the *S. oralis* Uo5 type; the *S. infantis* DD18 *licD4* cluster also differed from that of *S. oralis* Uo5. All three species contain the genes for uptake and activation of exogenous choline (*licABC*) as well as for decoration of teichoic acids (*licD* homologs). A closer look revealed that one group (*S. mitis* DD26 and *S. oralis* DD16, DD17, DD20, and DD21) contained a *lic4* region where the homology of the *licD3* and *tacF* gene products to the *S. oralis* Uo5 proteins was much lower (Table S2). This suggests that at least four biochemical variants of choline-containing teichoic acids occur in *S. mitis*, *S. oralis*, and *S. infantis*. The results are in agreement with data obtained by Kilian et al. showing that monoclonal antibodies directed against the backbone and the phosphocholine residues of TAs react with some strains of these three species (6).

### CBPs.

Choline-binding proteins (CBPs) are anchored to the cell wall by hydrophobic interactions with choline-containing teichoic acids (for a review, see reference 43). They are composed of a choline-binding module consisting of repeats of 20 amino acids and a nonconserved functional domain. They represent a highly varied family with respect to non-CBP modules, and numbers of CBPs also vary largely even within one species.

There are only three CBPs common to *S. mitis* B6, *S. oralis* Uo5, and *S. pneumoniae*, namely, LytB, a key enzyme for cell separation (44), CbpD, a murein hydrolase implicated in the lysis of noncompetent genes (45), and CbpF, a putative modulator of cell wall hydrolases (46), strongly suggesting that these CBPs have an important physiological role in these species. Genes encoding these three CBPs were found in the *S. oralis*, *S. mitis*, and the *S. infantis* genomes, which contained the *lic* clusters described above. All streptococcal genomes that did not contain CBPs, and thus did not contain CbpD, encoded a protein related to LytF of *S. gordonii* and possessing a similar function (47, 48) or another, new autolysin with a related CHAP domain (*Streptococcus* sp. strains DD10 and DD13).

### Noncoding csRNAs 1 to 6.

Streptococci contain a two-component regulatory system, CiaRH, which was originally identified in and characterized for *S. pneumoniae* (49–51). The system affects diverse processes, such as genetic competence (52, 53), bacteriocin production (54), host colonization (55), virulence (56), and β-lactam resistance (49, 57). Within the CiaR regulon, there are variable numbers of genes specifying small noncoding RNAs, i.e., *cia*-dependent small RNAs (csRNAs), ranging in size from 51 to 200 nucleotides (59). Since the csRNAs are involved in major CiaR-associated phenotypes in *S. pneumoniae* (53, 58), it was of interest to look for csRNA genes in the novel genomes.

First, the genomes were searched for the response regulator CiaR, which was clearly detected in all genomes with the typical recognition helix described previously (59). The corresponding histidine kinase, CiaH, was also present and showed a greater variability than CiaR, consistent with an earlier observation (59). Subsequently, the genomes of *S. mitis*, *S. oralis*, *S. gallolyticus*, and *S. gordonii* strains were searched for the types of csRNAs previously defined in other strains of these species (59). All csRNAs were present in the new strains. Interestingly, some *S. oralis* strains contained six instead of the five csRNAs of *S. oralis* Uo5 (17) caused by duplications of csRNA2, csRNA4, or csRNA6 genes. Two species with unknown repertoires of csRNAs contained csRNAs known from other species. *S. lutetiensis* harbored the *S. gallolyticus* UCN34 csRNAs except for csRNA40 (59), and *S*. *infantis* harbored four of the five *S. oralis* Uo5 csRNAs but not csRNA1. The other streptococci, especially those without species designation, did not yield full-length hits in the BLAST analysis with csRNA types defined by Marx et al. (59), indicating the existence of novel csRNAs in these bacteria.

Closer inspection of *S. oralis* sequences with duplicated csRNA genes revealed a surprising result. In between duplicated csRNA2 and csRNA6 genes (DD05 and DD15), we found a genetic island of four genes encoding redox proteins related to succinate dehydrogenase and fumarate reductase, a transporter of the oxalate/formate antiporter family and an AraC-type regulator. These genes are not present in *S. oralis* strains without duplicated csRNA genes. It appears therefore, that this small metabolic island is integrated into the *S. oralis* genome via csRNA genes. Similarly, an even smaller island of two genes encoding proteins without assigned functions is integrated between duplicated csRNA4 genes (DD27).

In *S. infantis* DD18, the four-gene island of *S. oralis* DD05/DD15 is integrated between two csRNA6 genes. A phage is apparently integrated into csRNA2 in DD14, but we could not deduce whether this is also related to a csRNA gene duplication due to termination of the contig sequence.

### *S. pneumoniae* virulence factors in viridans streptococci.

A large number of surface components important for the interaction with host cells have been described to occur in *S. pneumoniae* (for reviews, see references 60 and 61). Most of these genes were present in all genomes of this study, as has been described for *S. mitis* B6 (3), including the lipoprotein PsaA, a manganese transporter, and the two peptidyl-prolyl isomerases SlrA and PpmA, as were the nonclassical cell surface proteins, the plasminogen-binding proteins GAPDH (glyceraldehyde-3-phosphate dehydrogenase) and enolase, and the fibronectin-binding protein PavA. PavA is essential for colonization in the upper respiratory tract but probably mediates adherence indirectly by affecting other virulence factors (62, 63). The high conservation of PavA is exemplified in Fig. S3. Despite a high degree of sequence identity, every genome contained a distinct predicted PavA which differed from *S. pneumoniae* PavA by up to 3.6% (*S. mitis*) and 5.4% (*S. oralis*). In this context, it is interesting that none of the viridans streptococci investigated here contained the gene cluster implicated in riboflavin biosynthesis (*S. pneumoniae* R6 spr0161 to spr1064) except the *S. gallolyticus* and *S. lutetiensis* genomes. In contrast, the thiamine cluster absent in *S. mitis* B6 (3) was variably present in several *S. oralis* genomes (see Table S2 in the supplemental material).

10.1128/mSphere.00041-15.3 Figure S3  Phylogenetic tree of PavA in *S. oralis*, *S. mitis*, and *S. pneumoniae*. (A) The radial tree was generated with MEGA6 using Clustal alignment. PavA proteins from *S. pneumoniae* (R6) Hu663_19A, Spain 23F-1, and TIGR4 were included for comparison. Light blue, *S. oralis*; light pink, *S. mitis*. The bar refers to genetic divergence as calculated by the MEGA software. (B) Amino acid alignment of PavA proteins. Shown are residues which differ from the reference sequence, *S. pneumoniae* R6; the amino acid position is indicated by vertical numbers on top. Download Figure S3, TIF file, 0.5 MB. Copyright © 2016 Denapaite et al. 2016 Denapaite et al. This content is distributed under the terms of the Creative Commons Attribution 4.0 International license.

**FIG 8:**
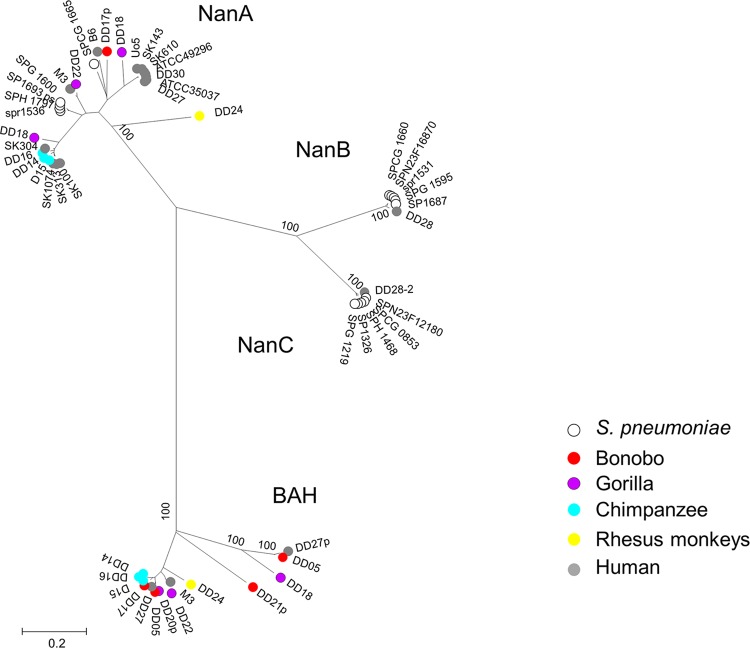
Neuraminidases and a conserved β-*N*-acetyl-hexosaminidase in *S. pneumoniae*, *S. mitis*, and *S. oralis*. The phylogenetic tree was generated with MEGA6 using the muscle alignment from the genomes used in this study and selected *S. pneumoniae* genomes. NanA, NanB, and NanC are indicated. p, partial sequences; BAH, putative β-*N*-acetyl-hexosaminidase. Bootstrap values (percentages) are based on 1,000 replications. The bar refers to genetic divergence as calculated by the MEGA software.

We investigated neuraminidases in more detail, since these enzymes target sialic acids, which differ between humans and primates. *N*-Acetylneuraminic acid (Neu5Ac) and its derivative *N*-glycolylneuraminic acid (Neu5Gc) are major sialic acids in many vertebrates, including the great apes. However, Neu5Gc is missing in human tissues due to an inactive form of the enzyme required for the generation of this compound (64, 65). In *S. pneumoniae*, three neuraminidases have been described: NanA, which contains an LPXTG motif, and NanB/NanC (for a review, see reference 4). NanA contributes to attachment to host cells by hydrolyzing terminal sialic acid residues from host proteins and polysaccharide components. The *S. mitis* genome of DD22 and many of the *S. oralis* genomes contained a closely related *nanA* homolog, as did the genomes of DD08 (*S. cristatus*), DD10 (unknown species), and DD04 (*S. sinensis*) (Table S2). In contrast, NanBC were absent in all *S. oralis* isolates and found only in one human *S. mitis* isolate, DD28 (Table S2). Instead, a protein encoding a β-*N*-acetyl-hexosaminidase occurred in most oral streptococci (Table S2), which was absent in all *S. pneumoniae* genomes. Like NanA, it contains a YSIRK signal peptide and represents an LPXTG cell surface protein. No primate-specific clustering was observed (Fig. 8).

A few genes described as *S. pneumoniae* virulence factors were not detected in the *S. mitis* B6 (3) or the *S. oralis* Uo5 (28) genome. This includes the three CBPs PspA, PcpA, and PspC (for reviews, see references 60 and 66), the hyaluronidase HysA, and the *cps* cluster responsible for the highly variable polysaccharide capsule. As shown recently, *cps* clusters have been imported from numerous *Streptococcus* species (5) and were not investigated here. Hyaluronidase activity is present in most *S. pneumoniae* isolates. It has been found in some *S. oralis*, but not in *S. mitis*, strains (6). Consistently with this observation, only one *S. oralis* genome harbored a HysA gene (DD25) (Table S2).

PspA is a highly immunogenic protein, and antibodies against PspA protected mice when challenged with *S. pneumoniae* (60, 66). It interferes with complement activation and is able to bind lactoferrin (67, 68). PspA sequences are highly divergent in *S. pneumoniae* due to intragenic recombination similar to that of PspC (60, 69). PspA has a mosaic structure in its central highly charged and proline-rich regions. BLAST analysis performed with the non-choline-binding domains revealed that only in the human isolate *S. mitis* DD28 is a PspA-related deduced protein identical to *S. mitis* B6 CBP2 (smi0038) present. N- and C-terminal sequences were closely related to *S. pneumoniae* PspA; however, the charged and proline-rich regions were distinct.

PcpA is conserved among pneumococci, and since it elicits protection in murine models of pneumonia and sepsis, it is now included in vaccination trials (70). The PcpA gene is associated with transposase elements, indicating acquisition from a still-unknown source. We found only in DD09 (*S. constellatus*) a PcpA homolog with 72% identity to *S. pneumoniae* PcpA (first 360 amino acids). However, it lacked the choline-binding domain and carried an LPXTG motif instead. We did not find evidence in other *S. constellatus* genomes for the presence of this gene, strongly suggesting that it is part of the accessory genome in this particular case.

*S. pneumoniae* PspC (also named CbpA) interacts with the secretory component of the polymeric immunoglobulin receptor and interacts with components of the innate immune system, such as the complement proteins C3 and factor H (71); it also binds to vitronectin (72). It is located on an island encoding TCS06 and an integral membrane protein of unknown function. Some isolates contain another PspC-like protein, but one which shows a C-terminal cell wall-anchoring LPXTG motif instead of the choline-binding repeat, and differ also in their proline-rich domains. PspC was also named Hic for factor H-binding inhibitor of complement (73, 74). PspC/Hic proteins are highly varied in *S. pneumoniae*, and only the signal peptide, as well as the overall domain organization, is conserved (74).

**FIG 9:**
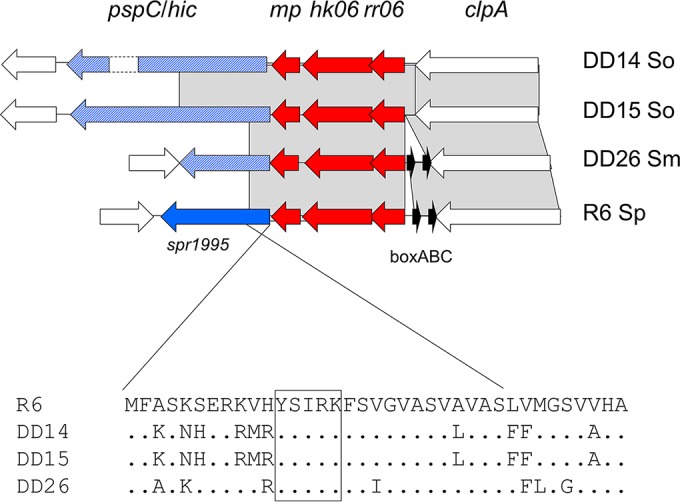
TCS06 islet. The genes encoding TCS06 and a conserved putative membrane protein (mp; red arrows) and a cell surface protein (blue arrow, PspC in *S. pneumoniae* [Sp] R6; blue hatched arrows, an LPXTG-containing protein in *S. mitis* [Sm] DD26 and *S. oralis* [So] DD14 and DD15) are shown. White genes represent flanking regions. Gray areas mark BLASTn matches between the sequences. BoxABC elements are shown in black. The signal peptide of the proteins is shown below the diagram; the pentapeptide motif conserved in many cell surface proteins is boxed.

PspC homologs containing the highly conserved N-terminal signal peptide of *S. pneumoniae* PspC were found in *S. oralis* DD14 and DD15 as well as in *S. mitis* DD26, encoded by a gene located in the same genetic environment as in *S. pneumoniae*, downstream of a TCS06 homolog (Fig. 9). However, as with Hic, these proteins contained an LPXTG motif and no choline-binding module and differed largely in their proline-rich internal regions; both *S. oralis* proteins were almost identical throughout the first 319 residues. It should be noted that the *pspC* island in *S. pneumoniae* is frequently associated with BOX elements (strain R6) or transposases (strain Hungary 19A_6) which may be involved in the variability of this region. Such elements were missing in the *S. oralis* pspC islands, and only two BoxABC elements were present in the *S. mitis* DD26 genome.

### LytA autolysin and Ply pneumolysin.

In contrast to all other CBPs, LytA does not contain a signal peptide and is therefore located mainly in the cytoplasm of the cells (75–77). It is still unclear how it accesses the pneumococcal cell wall, and it has been suggested that its activity is restricted to sites of nascent peptidoglycan biosynthesis (78). LytA encodes the major autolysin in *S. pneumoniae* and is responsible for stationary-phase lysis of pneumococcal cultures and for the lytic response to β-lactams and other cell wall inhibitors. It acts during genetic competence to lyse noncompetent cells, a process named “fractricide” (79), and it is probably required for the release of virulence factors, including the pneumolysin Ply (80). In *S. pneumoniae*, the LytA gene is located on a genomic islet, including the Ply gene, and has been imported probably via recombination with phages, which frequently carry a *lytA* homolog (81). LytA genes associated with cryptic phage relicts are genetically distinct. The presence of *lytA* and *ply* in *S. mitis* has been documented (3, 6, 81–83), but their genomic organization has not been elucidated.

**FIG 10:**
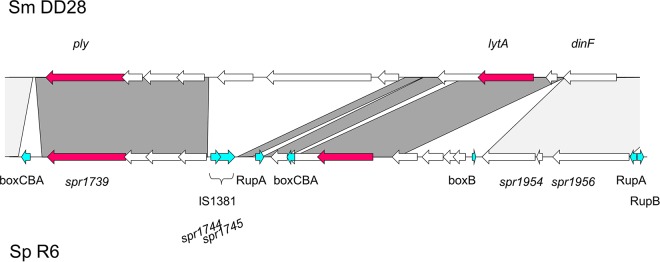
*ply*-*lytA* island in *S. mitis* DD28. Shown is a comparison of the region containing *ply* and *lytA* between *S. mitis* DD28 and *S. pneumoniae* R6. The genes *ply* and *lytA* are shown as red arrows. Dark-gray areas mark BLASTn matches between the two regions; light gray marks regions conserved in other strains of these species. Turquoise arrows, Rup, BOX, and IS elements.

LytA homologs were found in the genomes of three *S. oralis* isolates from primates. LytA from a wild chimpanzee was closely related to LytA from a zoo ape. Two human *S. mitis* isolates contained two copies, one of which was associated with phage genes, whereas the other one was located downstream of *dinF*, as with *S. pneumoniae* lytA. Both *S. mitis* DD28 and DD26 also contained a *ply* homolog. The organization of *lytA*-*ply* in *S. mitis* DD28 was similar to that in *S. pneumoniae* but included two genes not present in *S. pneumoniae*, while *S. pneumoniae* contained multiple fragmented insertion sequence (IS) elements and other mobile sequences, such as RUP and BOX elements (Fig. 10). The rare occurrence of RUP elements among *S. mitis* strains has been noted (5). A similar organization can be deduced from the DD26 genome, but it contained a sequence gap between *lytA* and *ply* (not shown). This shows that the complete island is present also among the *S. mitis* strains from humans.

In summary, almost all genes associated with *S. pneumoniae* virulence were found in the primate isolates. However, a small set of genes encoding the PspC islet, HysA, NanBC, and Ply-LytA, present in most *S. pneumoniae* strains, were restricted to a few *S. oralis* and/or *S. mitis* genomes.

## DISCUSSION

### Species isolated from primates.

One part of this study was to see which primate species contained streptococci related to those that are commensals in human. Streptococci could be isolated from great apes, Old World monkeys (rhesus monkeys), and lemurs (ring-tailed lemurs and Verreaux’s sifaka) and included a wide variety of species of viridans streptococci according to MLSA and genomic analyses. No streptococci of the Mitis group of viridans streptococci could be isolated from lemurs from Madagascar; we found only *S. gallolyticus*, which belongs to the Bovis group. However, members of the Mitis group of streptococci were obtained only from monkeys and great apes, with *S. oralis* being the predominant species. None of our samples showed 16S rRNA identity to other species of the Mutans group, including *Streptococcus troglodytes*, *Streptococcus dentirousetti*, *Streptococcus downei*, and *Streptococcus macacae*, which have been isolated from plaques of chimpanzees obtained by brushing their teeth (84), probably due to the different methods used for sampling. The streptococci included novel species isolated from wild chimpanzees that we could not define by MLSA or 16S rRNA analysis, and other genes did not reveal matches with >90% homology (Table S3). These findings should be corroborated by microbiome analyses to reveal potential differences between the commensal floras of primates.

The genomes of three strains isolated from ring-tailed lemurs defined one strain, KG3c, closely related to *S. dysgalactiae* subsp. *equisimilis* and two strains of the Mitis group, *S. lutetiensis* DD06 and *S. sinensis* DD04. It was difficult to obtain MLSA sequences from other ring-tailed lemur isolates, and only sequences from *rpoA* and *pyk* could be obtained from another seven strains. Phylogenetic analysis showed that they formed an unidentified cluster between *S. sinensis* and *S. gordonii* (not shown). *S. oralis* strains were obtained only from Old World monkeys, most of which were located on branches distinct from those containing the main cluster of human isolates in the phylogenetic trees generated by MLST and MLSA. This strongly suggests that *S. oralis* had evolved in these animals prior to the origin of humans and that this species is part of the commensal flora at least of great apes. The finding that *S. oralis* is also associated with rhesus monkeys should be confirmed by screening of wild animals, since the possibility of transfer of strains from humans to animals held in captivity cannot be excluded. Taken together, the phylogenetic tree of viridans streptococci appears to parallel the evolution of primates. Obviously, more samples from free-living animals, including New World monkeys, are needed to gain a comprehensive view of the evolutionary history of streptococci, which are important commensals in these animals.

In this context, it is remarkable that antibiotic resistance phenotypes and the TetM resistance determinant were found only in isolates from zoo animals and in those from the German primate center, i.e., in an environment where these phenotypes are frequent among *Streptococcus* spp. but not in free-living animals. Nevertheless, the possibility of transfer from humans to wild animals cannot be excluded. The sampling of fruit wedges is a successful strategy to screen for bacteria and viruses (85), and a human-to-monkey transmission of *S. aureus* has been reported (86).

As pointed out before (5, 8), the highly diverse subclusters within the *S. mitis* cluster could not be distinguished by phenotypic properties, challenging the definition of species. This is also apparent if one considers the *S. oralis* lineages (Fig. 2), which are probably a reflection of the diversification in different nonhuman hosts. Remarkably, the newly defined species *S. tigurinus* (87) and *S. dentisani* (15) cluster among the organisms of the *S. oralis* subcluster of IgA protease-negative organisms and the previously defined *S. mitis* biovar 2 subcluster (Fig. 2A), respectively, challenging the definition of *S. oralis*. 16S rRNA sequences are varied within designated *S. oralis* isolates, and thus the species of isolates that cluster within the heterogeneous *S. oralis* cluster, including the genomic information of many more strains, should be confirmed by further analyses. Determination to the species level is aggravated by the capacity for genetic transformation in viridans streptococci. The large accessory genome bears many signs of interspecies gene transfer, including large genomic islands common among different streptococcal species, leading to a smooth transition between species in comparative genomic hybridization experiments of oral streptococci (3, 4, 88). In this context, it is remarkable that csRNA genes apparently serve as entry sites for horizontal gene transfer in several cases, as described here, thereby contributing to the genomic variability observed for *S. oralis* and resulting in an overlap in the accessory genomes of *S. oralis* and *S. infantis*. It will be interesting to see if csRNAs with inserts are also found or are found even more often in other streptococcal species.

### Genome analysis of peptidoglycan and teichoic acid biosynthesis.

The second part of this study investigated cell surface components, including enzymes involved in cell wall polysaccharide biosynthesis, extending previous genomic analyses that focused mainly on *S. mitis* (3, 5). The variability of genes involved in peptidoglycan biosynthesis among *S. oralis* strains—those for PBPs and MurMN—is astounding. A high variability of PBPs in *S. mitis* is well known and has been exemplified recently using a large number of isolates (30). We now provide evidence that *S. oralis* isolates also differ largely in sequences encoding PBP2x, PBP2b, and PBP1a, proteins implicated in β-lactam resistance, and that these proteins are known to have a mosaic structure in resistant isolates. Mosaic blocks present in a common class of resistant mosaic PBP2x genes that are closely related to PBP2x genes from sensitive *S. mitis* isolates were found only in human isolates with high-level resistance (Fig. 7; see Fig. S2 in the supplemental material). In contrast, PBP2a sequences were conserved throughout the sequences (not shown). In several genomes, two genes encoding CPase PBP3 homologs, termed group 1 and group 2 CPases, were present (Fig. 6). In most cases, only the group 2 CPase gene encoded a protein with conserved active-site motifs. It is likely that that the products of these genes represent the only functional CPase. The gene encoding group 1 CPases was located in the same genomic environment, whereas the genes encoding group 2 CPases were present at locations in DD04 (*S. sinensis*), DD08 (*S. cristatus*), and DD07 (*S. gordonii*) that were distinct from those in DD09 (*S. constellatus*), DD11 (unknown species), and DD19 (*S. parasanguinis*); the genes in DD10 (unknown species) were again positioned differently, suggesting that these genes have been imported into the genomes on different occasions. It would be interesting to see whether the enzymatic activities of group 2 enzymes differ from those of group 1 enzymes.

*S. oralis* Uo5 lacks MurN, associated with an interpeptide bridge consisting of only one l-Ala residue (34), and we now show that *murM* and *murN* are also apparently lacking in some isolates (see Table S2 in the supplemental material). Accordingly, these strains most likely contain no interpeptide bridges in their peptidoglycan, which should be confirmed by biochemical analyses. This raises the question of which of the PBPs is preferentially affected by an altered substrate, a question that can be clarified only by complex genetic or biochemical experiments. Jensen et al. also noted the absence of MurM homologs in *S. mitis* and *S. oralis* (30) and hypothesized that this genotype is tolerated only in penicillin-sensitive strains. Interestingly, we found mutations in PBPs associated with resistance (Q552E in PBP2x of DD20 and T446A in PBP2b of DD24) in *S. oralis* isolates where both *murM* and *murN* were missing. Since deletion of MurMN in penicillin-resistant strains leads to a breakdown of resistance, including cefotaxime resistance, which is mediated by PBP2x but not by PBP2b, it has been speculated that it is the altered “resistant” PBP2x whose function depends on the presence of branched peptides (89). Given the variability of PBP sequences and of PBP2x in particular, it is quite possible that resistant PBP variants that are still functional even in the absence of MurMN have evolved. However, it might be difficult to find such isolates. Since resistant PBPs evolved in the genomic context of the respective *murMN* constellation and are transmitted mainly by gene transfer, resistant *de novo* variants of strains that do not contain *murMN* might be encountered only on rare occasions.

The variation observed in *S. oralis* strains with respect to teichoic acid biosynthesis clusters responsible for choline decoration of teichoic acids (LTA type IV) is astounding. All strains contained genes required for choline-containing TAs, but the presence of distinct *lic* clusters (*lic3* versus *lic4* of Uo5 and *lic4* of *S. oralis* cluster 2 isolates) strongly suggests three different biochemical makeups of this cell surface polysaccharide in *S. oralis* strains and in at least two variants of *S. mitis*. Kilian et al., using monoclonal antibodies to detect epitopes characteristic of the backbone and the phosphocholine residues of the TA, showed that *S. infantis* contains choline in its cell wall (6). We now provide genetic evidence for the presence of these components in *S. infantis*, with DD18 containing a *licD4* cluster similar to the *licD4* cluster in Uo5; more *S. infantis* genomes are needed to confirm that *lic* genes and CBP genes are uniformly present in this species and whether variants occur, as shown here for *S. oralis*. All species with type IV LTAs share the physiologically important choline-binding proteins CbpD, CbpF, and LytB. The other viridans streptococci investigated here contain LtaS synthase to polymerize a much simpler LTA (type I) consisting of a polyglycerolphosphate chain (39). Some *S. mitis* strains contain *ltaS* in addition to the *lic* operons, similar to what occurs in *S. mitis* B6 (40). As pointed out before, experimental evidence is required to know whether these strains express two types of LTA.

### *S. pneumoniae* virulence factors.

In general, this study confirmed that many genes encoding so-called virulence factors of *S. pneumoniae* are present in many strains among viridans streptococci, as has been shown in several genomic studies (3, 5, 6, 28). We focused our analysis on cell surface proteins since these are the components that interact with host cells and thus are potential candidates to reveal differences between *S. pneumoniae* and related streptococci. The main finding was that the neuraminidases NanBC, which are variably present in *S. pneumoniae* genomes, were found only in one *S. mitis* isolate and were completely absent in *S. oralis* and other viridans streptococci. In contrast, a related protein with predicted *N*-acetyl-hexosaminidase activity occurred in all *S. oralis* genomes, independently of the host of the isolates, and was present also in the *S. infantis* genome. The *in vivo* role of this protein remains to be clarified. We failed to detect features that are exclusively associated with primate versus human isolates for several reasons. First of all, the sample size for one streptococcal species from a single primate species is still too small to interpret results reliably in this respect. It is possible that the capsule plays an important role for host specificity, as has been pointed out for *S. pneumoniae* (5), but due to the variability of capsular clusters, it is difficult to interpret the variability encountered, e.g., in *S. oralis* genomes. Also, differences that are due to host specificity might not be visible at the genomic level but require physiological tests or biochemical analyses (e.g., tests for the glycosylation pattern of surface components).

Two clusters which included *S. pneumoniae*-specific virulence genes were found among *S. mitis* and *S. oralis*: the *ply* (or *lytA*) gene cluster and the TCS06 *pspC* islet. The presence of *ply*-*lytA* in commensal streptococci is well known (3, 6, 81–83), but this is the first time that we can show that the entire island is present in some *S. mitis* strains and that it is located at the same genomic position as in *S. pneumoniae*. The main difference is the absence of the repeat elements RUP and BOX (Fig. 10). RUP elements have apparently undergone extensive expansion during the evolution of *S. pneumoniae* (90), whereas they are rarely found in *S. mitis* or *S. oralis* (3, 5). Similarly, the TCS06 *pspC* cluster (Fig. 9) includes BoxABC elements in *S. pneumoniae* which were missing in the two *S. oralis* genomes containing this islet; they were present in the *S. mitis* genome. Generally, BOX elements are much rarer in *S. oralis* than in *S. mitis* or *S. pneumoniae*. Sequences related to the three novel *S. mitis*/*S. oralis* PspC-like proteins were found in *S. pneumoniae* genomes (e.g., strains NT_110_58 and Hungary 19A_6), documenting a remarkable example of domain shuffling and protein diversification during evolution.

There are several open questions that remain. What is it that makes *S. pneumoniae* a pathogen? Do the *S. oralis*/*S. mitis* strains that contain PspC, HysA, and the *lytA*-*ply* island have a higher-pathogenicity potential than those that lack these components? Is it the combination of these well-known virulence genes plus PcpA, PspA, and the highly variable polysaccharide capsule (which are present in most pneumococcal strains) what imparts pathogenicity? What is the role of the *N-*acetyl-hexosaminidase in *S. oralis* and *S. infantis*? What is the driving force behind the variation observed in peptidoglycan and the teichoic acid biosynthesis enzymes, PBPs, MurMN, and LicD3/4? Are there host-specific components that occur in human as well as in primate isolates? The speed of genomic research and novel biochemical tools might help to solve some of these riddles.

## MATERIALS AND METHODS

### Bacterial strains.

Swabs were obtained from the Frankfurt Zoo (throat swabs from bonobos, orangs, and gorillas) and from the German Primate Center, Göttingen, Germany (throat swabs from rhesus monkeys and nose swabs from ring-tailed lemurs). Throat swabs from free-living lemurs (Verreaux’s sifakas, *Propithecus verreauxii*; red-fronted lemur, *Eulemur rufifrons*; Western fat-tailed dwarf lemur, *Cheirogaleus medius*; gray mouse lemur, *Microcebus murinus*) in the Kirindy Forest in Madagascar, which is part of a field site operated by the German Primate Center, were obtained during a survey of anesthetized animals in the course of an annual marking and survey mission that followed the protocol described previously (91). Samples from wild chimpanzees from the Taï National Park, Ivory Coast, where contact to humans is highly restricted, were isolated from fruit wedges containing the fruit of two species of plants (memecylon and Parinari), which are chewed by the animals for long time periods and sucked on intensively before they are spit out. These fruit wedges where collected once the chimpanzees had reached a minimum distance of 10 m from the sample, which was placed in STGG medium (92) and transported to the field camp, where they were preserved in liquid nitrogen and shipped to Germany as described previously (86). Samples were vortexed and streaked on blood agar plates using a 10-µl inoculation loop. Plates were incubated overnight, and colonies showing alpha-hemolysis were isolated and tested for optochin susceptibility (see Table S1 in the supplemental material). Bacterial samples from swabs were grown in C medium (93) supplemented with 0.1% yeast extract, diluted, and streaked on d-agar plates (94) with 3% defibrinated sheep blood. Individual colonies suspected of representing viridans streptococci were isolated, and antibiotic susceptibility was tested with the Etest (β-lactam antibiotics) and antibiotic discs (all other antibiotics) (Table S1).

### Bacterial genomes.

The 25 genomes of isolates from primates and their accession numbers are listed in Table S1 in the supplemental material. In addition, seven genomes from human *S. mitis* and *S. oralis* isolates which were used in previous studies (10) were included for comparison (Table S1). Files with sequence reads from 454 3K paired-end sequencing technology were available for 26 strains isolated from various monkeys and monkey groups, including 24 from *Streptococcus* spp. The gsAssembler (Newbler), version 2.6, from Roche was applied for assembly. The rapid annotation subsystem technology (RAST) server (95) designed for annotation of bacterial and archaeal genomes was applied to obtain EMBL-formatted files containing protein, tRNA, and rRNA annotations from a large set of several output formats; *S. mitis* NCTC10712 was annotated by best BLAST analysis (96).

### DNA isolation and PCR amplification.

Chromosomal DNAs from streptococci were isolated as described previously (97). PCR products were purified using a JetQuick DNA purification kit (GenoMed). PCRs were performed using either Goldstar Red *Taq* polymerase (Eurogentec) or DreamTaq polymerase (Fermentas) according to the manufacturer’s instructions. The oligonucleotides used in this study were obtained from Eurofins. PBP2x gene fragments were amplified with the primers pn2xup and pn2xdown, as described previously (97). 16S rRNA sequences were amplified by PCR with the bacterium-specific primers rRNA2 (TCAGATTGAACGCTGGCGGC) and rRNA1 (TATTACCGCGGCTGCTGGCA) or the *Streptococcus* sp.-specific primer rRNA-Strep1rev (CTTACGGTTACCTCACCGACTTCG) and rRNA2.

### Identification of csRNA genes.

The genomes were searched for csRNA genes by BLAST analysis using the genes of 40 csRNA types defined by Marx et al. (59) as a query. Hits covering at least 50 consecutive base pairs were taken, and their genomic upstream regions were visually inspected for the presence of a typical CiaR-regulated promoter with the CiaR-binding site NTTAAG-5-TTTAAG placed 10 bp upstream of a −10 region. In all cases, such a promoter sequence was identified. It allowed us to predict exactly the start of the csRNA genes. Subsequently, the last T in the terminator region was taken to define csRNA genes completely.

### Bioinformatic tools and analysis.

BLAST searches were performed using the NCBI microbial genome data bank. A specialized search for the primate genomes was established on the NBC11 bioinformatic computational site http://nbc11.biologie.uni-kl.de/ (database searches/BLAST primate isolates) for all contigs. Neighbor-joining trees were generated with MEGA6.06 (98) and Clustal alignments using standard parameters; in some cases, muscle alignment was chosen, as stated in the text. Bootstrap analysis was based on 1,000 replicates; for MLST-derived phylogenetic trees, 500 replicates were used. For comparison, analyses were also conducted with the neighbor-joining algorithm.

### Nucleotide sequence accession numbers.

The whole-genome shotgun project sequences have been deposited in DDBJ/EMBL/GenBank (accession numbers are listed in Table S1 in the supplemental material). The versions described in this paper are versions XXXX01000000. The accession numbers for 16S rRNA sequences are listed in Table S4. Primers used for PCR amplification of internal gene sequences that were used for MLST and MLSA have been published (8, 10). Accession numbers for reference MLST genes (10) are EU075657 to EU076239. The accession numbers of MLST/MLSA sequences generated in this study are listed in Table S4.

10.1128/mSphere.00041-15.7 Table S4  Accession numbers for MLSA/MLST and 16S rRNA gene sequences. Download Table S4, XLSX file, 0.03 MB. Copyright © 2016 Denapaite et al. 2016 Denapaite et al. This content is distributed under the terms of the Creative Commons Attribution 4.0 International license.
