# Supplementary figures and images for: Highly Variable Streptococcus oralis Strains Are Common among Viridans Streptococci Isolated from Primates
Source: mSphere. 2016 Mar 9;1(2):e00041-15. doi: 10.1128/mSphere.00041-15 (PMC4863584; doi:10.1128/mSphere.00041-15)

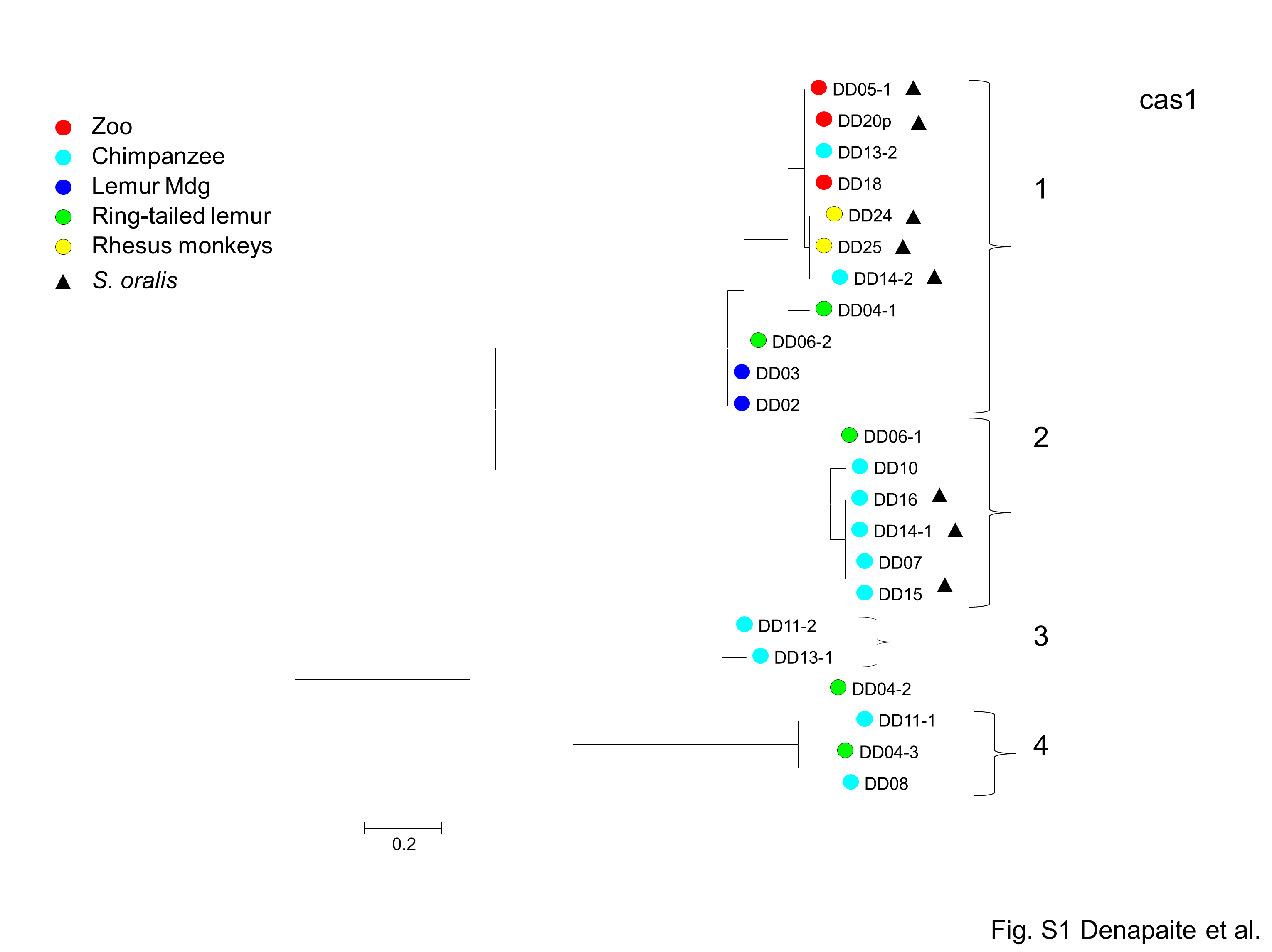

Supplement: Figure S1 [file sph002162037sf1.tif]

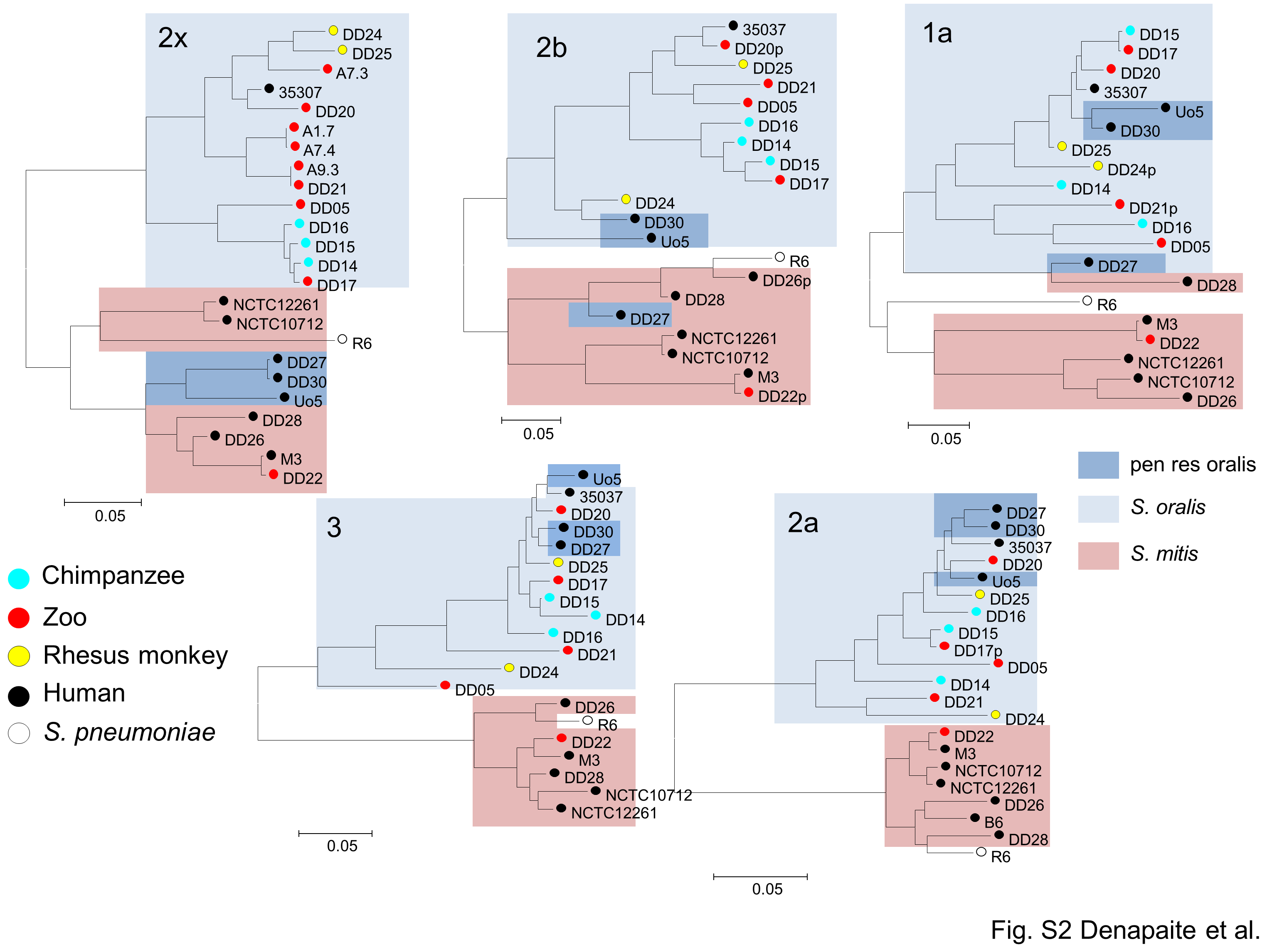

Supplement: Figure S2 [file sph002162037sf2.tif]

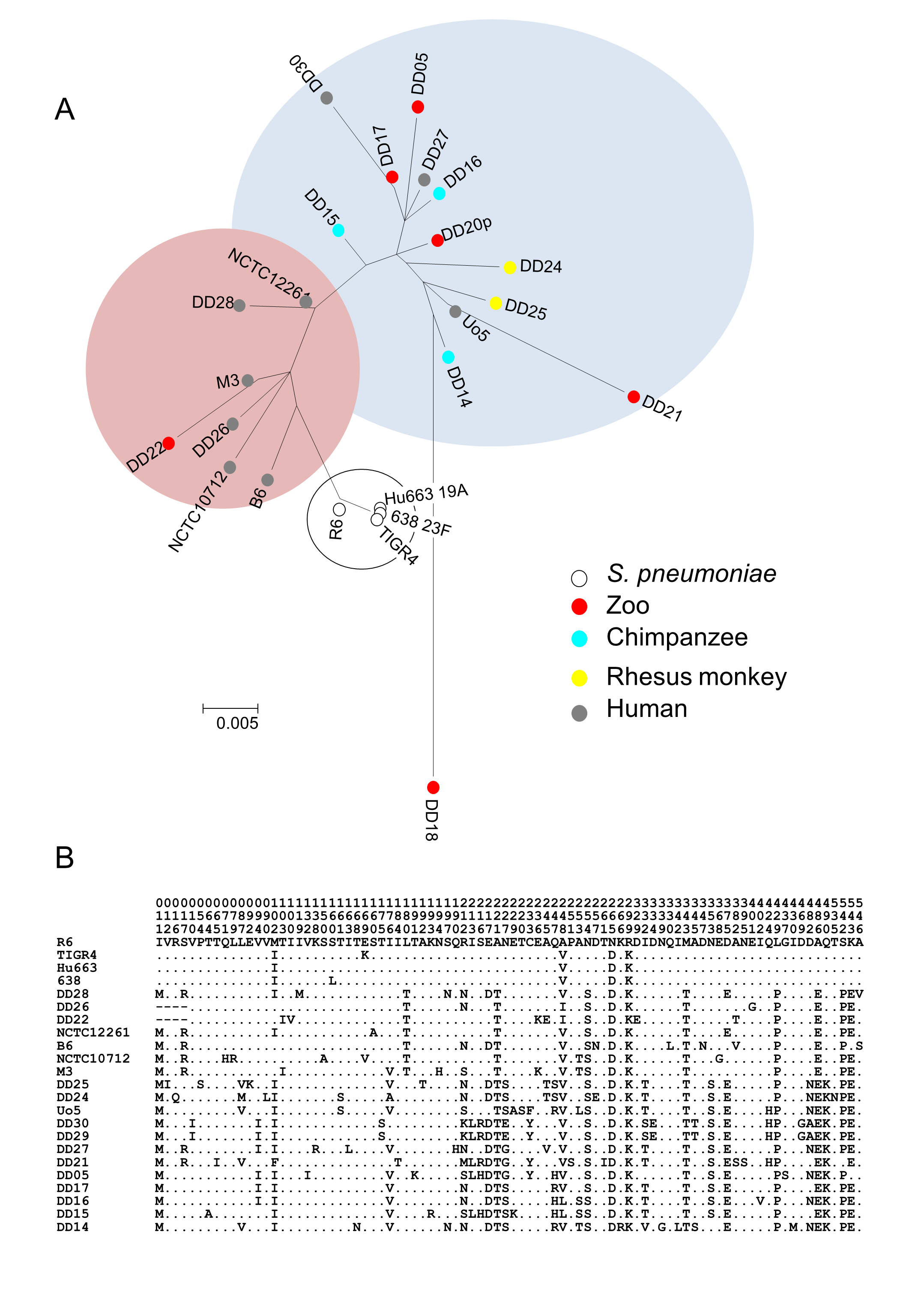

Supplement: Figure S3 [file sph002162037sf3.tif]
